# Comparative study of TiO_2_–Fe_3_O_4_ photocatalysts synthesized by conventional and microwave methods for metronidazole removal

**DOI:** 10.1038/s41598-023-39342-9

**Published:** 2023-07-26

**Authors:** Adam Kubiak

**Affiliations:** Faculty of Chemistry, Adam Mickiewicz University, Uniwersytetu Poznanskiego 8, 61614 Poznan, Poland

**Keywords:** Materials chemistry, Catalysis, Photocatalysis

## Abstract

This study focused on a direct comparison of conventional hydrothermal and microwave treatment during the synthesis of TiO_2_–Fe_3_O_4_ photocatalyst, which is an effective catalyst for decomposing metronidazole. The photocatalyst underwent various characterization analyses, including X-ray diffraction, Raman spectroscopy, transmission electron microscopy, energy dispersive X-ray, and diffuse reflectance spectroscopy. The Raman spectroscopy analysis revealed that the materials obtained through the conventional hydrothermal treatment consisted of separate phases of anatase and magnetite. On the other hand, the materials synthesized using the microwave process showed a noticeable shift in the E_g_ band (143 cm^−1^) and its half-width towards higher wavenumbers. This shift is likely due to the introduction of Fe ions into the TiO_2_ lattice. Additionally, both conventional hydrothermal and microwave synthesis routes produced TiO_2_–Fe_3_O_4_ systems with superparamagnetic properties, as demonstrated by SQUID magnetic measurements. The TEM analysis revealed that the materials synthesized using the microwave process exhibited higher homogeneity, with no noticeable large aggregates observed. Finally, this work proposed a convenient LED photoreactor that effectively utilized the photo-oxidative properties of TiO_2_–Fe_3_O_4_ photocatalysts to remove metronidazole. Combining photoactive TiO_2_–Fe_3_O_4_ catalysts with an energy-efficient LED reactor resulted in a low electrical energy per order (E_EO_).

## Introduction

In the current global situation, it is extremely important to care for the natural environment in the pursuit of climate neutrality. This objective is at the heart of the *European Green Deal*^1,2^, which has been accepted by all countries in the European Union. However, it is also crucial to skillfully use available energy resources. Now more than ever, the world sees that changing our energy strategy will achieve climate neutrality and ensure peace of mind in the world’s electricity markets. Additionally, researchers are constantly challenged to consider more environmentally friendly methods for the fabrication of desired products^3^. Among the guiding principles of green chemistry, the desire to use safer solvents and design for energy efficiency are two key principles relevant to materials science^4^.

Titanium dioxide is one of the most extensively studied powder materials in materials science, with nearly 200,000 results in the Scopus database (access date May 10, 2023). Its popularity can be attributed to its excellent photocatalytic properties, which make it suitable for the photooxidation of organic pollutants. However, the use of titanium dioxide in industrial wastewater treatment is limited due to certain drawbacks^5,6^. One such limitation is the difficulty in separating TiO_2_ from the post-process mixture. Another is the high rate of recombination of electron/hole charge carriers, which reduces process efficiency over time^7^. This affects the overall process performance and requires significant investments in TiO_2_ suspension separation to recover the photocatalyst for reuse in subsequent processes. One possible strategy to address the challenges of titanium dioxide photocatalysts is to incorporate a component that can improve material separation after the process^8,9^. Magnetite (Fe_3_O_4_), which is a mixture of two iron oxides has ferromagnetic properties due to the unbalanced magnetic contributions of Fe^II^ and Fe^III^ electrons^10^. The combination of the properties of both oxides allows for the production of magnetic photocatalysts for use in the photodegradation of organic pollutants. For instance, Chu et al.^11^ synthesized a stable core–shell TiO_2_@Fe_3_O_4_ system based on carbon, which exhibited enhanced photocatalytic capabilities. Similarly, Guo et al.^12^ developed a TiO_2_/Fe_3_O_4_/graphene material with increased activity for the removal of methylene blue. However, selecting an appropriate synthesis method that preserves both the photooxidative capacity and magnetic properties of the final system remains a major challenge for researchers^13^.

Currently, one of the most popular processes in materials science is the sol–gel technique, which is based on the hydrolysis reaction of organometallic precursors in the reaction mixture^14^. However, one of the main disadvantages of this process is the need for additional heat treatment at high temperatures, which requires significant amounts of electricity^15^. Additionally, the process generates off-gases that contribute to the carbon footprint^16,17^. Another popular process is the hydrothermal method, which simulates the growth of crystals during the mineralization process in nature^18^. This process is based on conventional heating, which heats the walls of the reactor and then the reactants by convection or conduction^19^. The reactor acts as an intermediary for the transfer of thermal energy from an external heat source, and this path typically leads to thermal gradients in the bulk media and to inefficient and heterogeneous reactions. Moreover, the undoubted disadvantages of the hydrothermal method include high equipment requirements, long reaction time, and high energy consumption^20,21^. However, taking into account the mild conditions of hydrothermal processes, pioneering work of changing the heating mechanism by including microwaves was carried out by Komarneni and Roy^22^. The authors reported that microwave heating involves two main mechanisms, dipolar polarization, and ionic conduction. Hence, microwaves heat any material containing mobile electric charges, such as polar molecules or conducting ions in a solvent or in a solid. Due to the high rate constant of the process, the microwave process can sometimes lead to overheating of the reaction mixture, which can lead to unwanted side reactions^23^. Moreover, the lack of precise temperature control can result in the formation of non-uniform products^24^. Despite this, microwave heating is indicated as an alternative heat source for rapid volumetric heating with a shorter reaction time and higher reaction rate. However, it seems that in the case of microwave-assisted processes, other effects occur in addition to shortening the processing time. Among others, Jiang et al.^25^ indicate the effect of microwave irradiation on nucleation relative to crystal growth. In addition, available scientific reports indicate that microwave radiation determines molecular processes due to differences in the absorption of microwave energy by compounds in the reaction mixture, which causes local temperature changes^26–28^. In another study, Teixeira et al.^5^ synthesized Na_2_Ti_6_O_13_ nanoparticles using the hydrothermal synthesis method with a lower concentration of NaOH under soft conditions. It is worth paying attention to the work presented by Gedye et al.^29^, who indicate the influence of many factors, such as sample volume, solvent, homogeneous and heterogeneous reactions, reaction vessel size, and power level on microwave-assisted processes. However, until today, the detailed mechanism of the chemical reaction under microwave irradiation has still not been fully understood^30,31^. Zhu and Cheng^32^ indicate that in many cases, reports on the specific microwave effects were based on inaccurate comparisons of microwave heating with conventional heating. Therefore, the authors reported that a large number of carefully designed comparative studies between microwave heating and conventional heating are necessary for further understanding of microwave heating mechanisms.

To meet the actual expectations, this paper compares the synthesis of TiO_2_–Fe_3_O_4_ photocatalyst using conventional hydrothermal and microwave treatment to meet current expectations. The novelty of this study is demonstrated by the different mechanisms used to produce the systems. The materials created through the microwave process exhibited a shift in the E_g_ band towards higher wavenumbers due to the introduction of Fe ions into the TiO_2_ lattice. Additionally, the study proposed an LED photoreactor that effectively utilized the photo-oxidative properties of TiO_2_–Fe_3_O_4_ photocatalysts to remove metronidazole, resulting in a low electrical energy per order (E_EO_). The improvement in photoactivity is attributed to the formation of a heterojunction between the TiO_2_ and Fe_3_O_4_ components of the system.

## Materials and method

### Materials

TiCl_4_ (97%), FeCl_2_·4H_2_O (98%), FeCl_3_·6H_2_O (97%) NH_3_·H_2_O (25%), PEG400 (p.a.), ammonium oxalate (99%), silver nitrate (>99%), tert-butyl alcohol (99%) benzoquinone (p.a.) and metronidazole (>99%) were purchased from Sigma-Aldrich (USA). The used reagents had an analytical grade. Whereas the water used in all experiments was deionized.

### Synthesis of TiO_2_–Fe_3_O_4_ systems

The synthesis of the TiO_2_–Fe_3_O_4_ systems was realized using the conventional hydrothermal and microwave routes. In the first stage, titanium(IV) chloride solution (1 wt%) was prepared in distilled water in an ice-water bath according to the procedure previously reported elsewhere^33^. The TiCl_4_ solution was then placed in an IKA reactor (Ika Werke GmBH, Germany) and stirred at 100 rpm. A 25% ammonia solution was then added with constant stirring until pH 9. The resulting suspension was transferred to a hydrothermal reactor (Parr Instrument Co., USA) or a microwave reactor (CEM Discover SPD 80, USA) and subjected to heat treatment. The heat treatment parameters were: for the hydrothermal process—T = 200 °C, t = 12 h, for the microwave treatment—T = 200 °C, t = 5 min, P = 300W. After the process was completed, the reactor was cooled to room temperature and the obtained material was washed three times with deionized water. Then it was dried at 60 °C for 6 h.

The procedure for obtaining TiO_2_–Fe_3_O_4_ systems was started by placing 50 mL of the mixture of Fe_3_O_4_ precursors in the ratio of Fe^2+^:Fe^3+^ = 1:1.5 (0.1 g:0.2 g) ions and a 0.1 g of polyethylene glycol in the IKA reactor (Ika Werke GmBH, Germany). Then, 3 mL of ammonia water solution was added to the reaction mixture, and the system was stirred at 1000 rpm. Subsequently, a specified amount of TiO_2_ suspension (see Table S1 in the Supplementary Materials) was added to the resulting mixture and homogenized for 30 min. The resulting reaction mixture was then subjected to hydrothermal microwave treatment. The heat treatment parameters were: conventional hydrothermal—T = 200 °C, t = 12 h, microwave treatment—T = 200 °C, t = 5 min, P = 300W. The resulting materials were separated using an external magnetic field and washed until a clear liquid was obtained over the layer of the system. The washed precipitate was dried at 45 °C for 12 h. Each of the synthesized samples series was described according to the following formula:

1 $$Method\;abbreviation\_X\% {\mathrm{Fe}}_{3} {\mathrm{O}}_{4}$$

where *Method abbreviation*—M in case the microwave processing; H—for conventional hydrothermal treatment. *X*%—the amount of magnetite (wt% Fe_3_O_4_ = 2.5%; 5%; 10%; 15% or 20%).

### Characterization of synthesized systems

The X-ray diffraction analysis was performed using a Rigaku Miniflex 600 diffractometer (Rigaku, Japan). The measurement was conducted using CuKα radiation (λ = 1.5406 Å) in the range of 2θ = 20°–80° at a scan speed of 1°/min. The International Center for Diffraction Data (ICDD) database was used to interpret the obtained diffractions. Quantitative analysis of the phase composition, including the standard deviation, was performed using the reference intensity ratio method with the most intense independent peak of each phase. The crystallite size of the analyzed materials was determined using the Scherrer equation (Eq. 2)^34,35^:

2 $$D = \frac{K\lambda }{{\beta cos\theta }}$$

where *D*—average crystallite size (nm); *K*—Scherrer constant (0.891); *λ*—X-ray wavelength (λ = 1.5406 Å); *β*—line broadening at half the maximum intensity (FWHM), *θ*—Bragg angle (degree).

Raman spectra were performed using the Renishaw micro-Raman system from inVia. The spectra were recorded in the spectral range of 100–1600 cm^−1^, in the backscattering geometry by the confocal. The excitation light of 488 nm was used by a tunable Ar-ion laser. Renishaw WiRE 3.4 software was used for data collection and deconvolution of the obtained spectra.

The morphology and microstructure of the obtained systems were determined using the Hitachi HT7700 transmission electron microscope (TEM) operating in high contrast and high-resolution mode. The maps of selected elements of the synthesized materials were generated using the same TEM (HT 7700) operating in scanning transmission electron microscopy (STEM) mode, equipped with the EDS X-ray microanalysis system from Thermo Scientific (USA).

The textural properties of surface area, pore volume, and pore diameter were determined using the low-temperature nitrogen sorption method. The ASAP 2020 porosimeter from Micromeritics Instrument Co. in Norcross, USA was used for the analysis. Prior to measurement, the materials were degassed at 120 °C for 4 h. The surface area was determined using the multipoint BET method by analyzing adsorption data in a relative pressure (*p*/*p*_0_) range of 0.05–0.30.

Magnetic measurements were carried out using the Quantum Design MPMS-XL SQUID magnetometer (USA). The temperature dependence of magnetization was measured in a magnetic field of 0.1 T over the temperature range of 2–300 K. Magnetization loops were collected at both 5 K and 300 K in magnetic fields up to 5 T.

Diffuse reflectance spectroscopy (DRS) was used to measure the light absorption properties of all obtained oxide materials. The measurements were conducted using a Thermo Scientific Evolution 220 spectrophotometer (Waltham, USA) equipped with a PIN-757 integrating sphere, and BaSO_4_ was used as a reference. The bandgap energy of the obtained samples was calculated from the plot of (F(R)·E)^0.5^ against E, where *E* is the photon energy, and *F*(*R*) is the Kubelka–Munk function proportional to the absorption of radiation.

The photoluminescence (PL) measurements were carried out using a spectrofluorometer (Fluorolog version-3 Horiba, Japan) with a 450 W high-pressure xenon arc lamp as an excitation source. The photoluminescence excitation (λ = 330 nm) and emission spectra were acquired at room temperature at a spectral resolution of 2 nm at a slit width of 2 mm.

The Jupiter STA 449 F3 apparatus (Netzsch GmbH, Bad Berneck im Fichtelgebirge, Germany) was used to determine thermogravimetric curves. The analysis were performed under flowing nitrogen at a heating rate of 10 °C/min in a temperature range of 30–1000 °C.

### Photocatalytic activity

Initially, a solution of metronidazole (20 mg L^−1^) was prepared in a volume of 1 L and mixed with 1 g of photocatalyst in the photoreactor. Exactly the same amount of metronidazole solution (1 L) was used for each experiment. To prevent the influence of indoor light, the process was conducted in a black box. The resulting mixture was homogenized in darkness for 30 min to establish adsorption/desorption equilibrium. Afterward, the UV-LED lamp was switched on, and the reaction mixture was irradiated. The detailed characteristics of the LED light source used are presented in Supplementary Materials. At intervals of 20 min (up to 180 min, after which the irradiation was stopped), 3 mL of the suspension was collected and filtered through a syringe filter (Macherey–Nagel, Germany). The filtered solution was analyzed using a UV–Vis spectrophotometer (UV 2020, Shimadzu, Japan) in the wavelength range of 200–700 nm, using the spectrum of demineralized water as the baseline. The maximum absorbance of pollution was observed at a wavenumber of 315 nm. The photocatalytic activity of the samples was determined by applying the calibration curve method, which had the formula y = 0.028x − 0.009, where x was the metronidazole concentration and y was the maximum absorbance value. The investigation aimed to comprehend how charge carriers and reactive oxygen species participate in the photocatalytic reaction, providing insights into the mechanism behind the degradation of organic contaminants when using the synthesized photocatalysts. To evaluate the photocatalytic activity, the procedure described above was followed, but with the inclusion of scavenger solutions in appropriate amounts. The concentrations of these scavenger solutions were adjusted to achieve a level of 20 mg/dm^3^ for metronidazole. Specifically, ammonium oxalate was selected as the scavenger for holes (h^+^), AgNO_3_ for electrons (e^-^), tert-butyl alcohol for free hydroxyl radicals (^*^OH), and benzoquinone for superoxide radical anions (^*^O_2_^–^).

## Results and discussions

### Crystal structure

**Figure 1:**
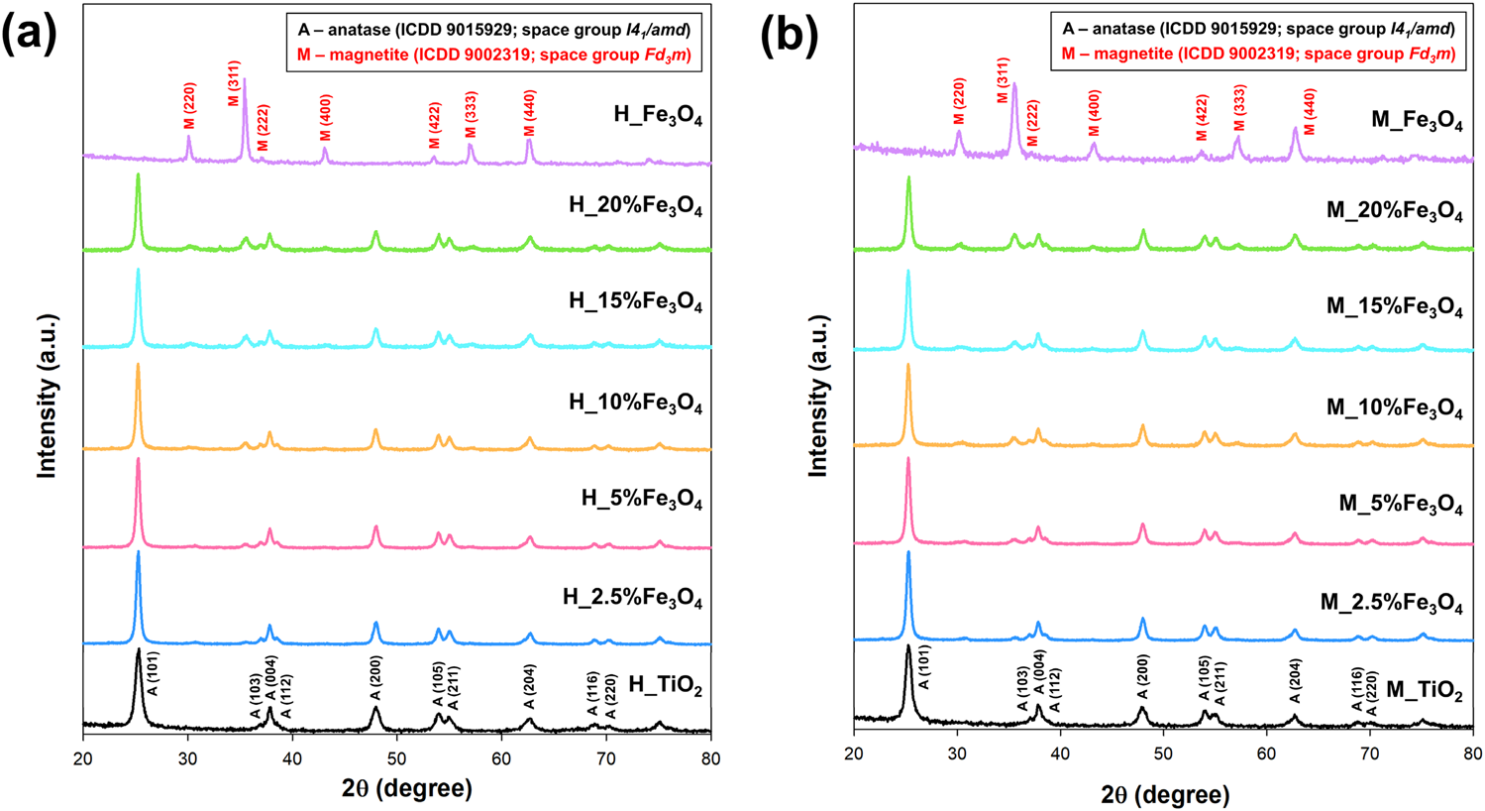
The XRD patterns for TiO_2_–Fe_3_O_4_ systems synthesized by (**a**) hydrothermal and (**b**) microwave methods.

The X-ray diffraction patterns of TiO_2_–Fe_3_O_4_ systems synthesized by conventional and microwave treatment are shown in Fig. 1. To determine the structural properties of the samples, Rietveld analysis was conducted using FullProf software, and the results for selected systems are presented in Fig. S1 of the Supplementary Materials. The phase composition and average crystallite size for the synthesized materials are summarized in Table S2 in Supplementary Materials. The black curves in the figure represent the typical XRD pattern of anatase^36,37^. The TiO_2_ reference materials obtained a similar crystallite size of 18.1 nm, regardless of the treatment method used (conventional hydrothermal or microwave). The diffraction peaks of the reference Fe_3_O_4_ standard^38,39^ were well indexed with relative intensities, indicating the as-synthesized nanoparticles were the pure Fe_3_O_4_ phase and single-phase of the face-centered-cubic spinel. The magnetite phase was confirmed for both conventional and microwave treatments. However, differences in the average size of crystallites for Fe_3_O_4_ were observed, with sizes of 21 nm and 19 nm for the hydrothermal and microwave procedures, respectively. XRD diffraction peaks from anatase and magnetite were observed regardless of the treatment method used. It is worth noting that the size of the anatase crystallites is close to the reference material for the TiO_2_–Fe_3_O_4_ systems synthesized by conventional heating, but the use of microwave treatment leads to a decrease in the size of the anatase crystallites. When analyzing the determined phase compositions, it should be noted that the use of conventional heating leads to the assumed values. In the case of the microwave method, a smaller share of the anatase phase was observed in the TiO_2_–Fe_3_O_4_ systems. The observed differences in the phase systems are related to the kinetics of the conventional and microwave processes. The influence of the process kinetics on the final material is also apparent in the clear differences in the values of the lattice parameters compared to the literature. According to the data presented by Kubiak et al.^37^, the lattice parameters for anatase are a = 3.785 Å and c = 9.514 Å, and for magnetite, c = 8.396 Å. While the values obtained by us for magnetite are close to the literature, the increase in the *a* and *b* parameters in the case of the anatase was shown. The similarity of the ionic radical of Ti^4+^ (0.604 Å) and Fe^3+^ (0.645 Å) suggests the possibility of occupying some titanium dioxide lattice sites by iron ions^40^, leading to a reduction in the crystallinity of the final material, and an increase in the lattice parameters of the unit cell. Kamani et al.^41^ have also suggested that the reduction in crystallite size of Fe-doped materials compared to undoped nanoparticles may be related to the transfer of a small amount of Fe ions to the interstitial or TiO_2_ substitution site^42^. Additionally, it is worth noting that the increase in the *a* and *b* lattice parameters for anatase is particularly visible for materials synthesized by microwave processing, while there are no clear differences for materials obtained by conventional hydrothermal heating. This confirms the earlier observations of scientists that the high kinetics of the microwave process may result in disturbances in the crystal lattice, which ultimately may lead to the introduction of dopants ions.

**Figure 2:**
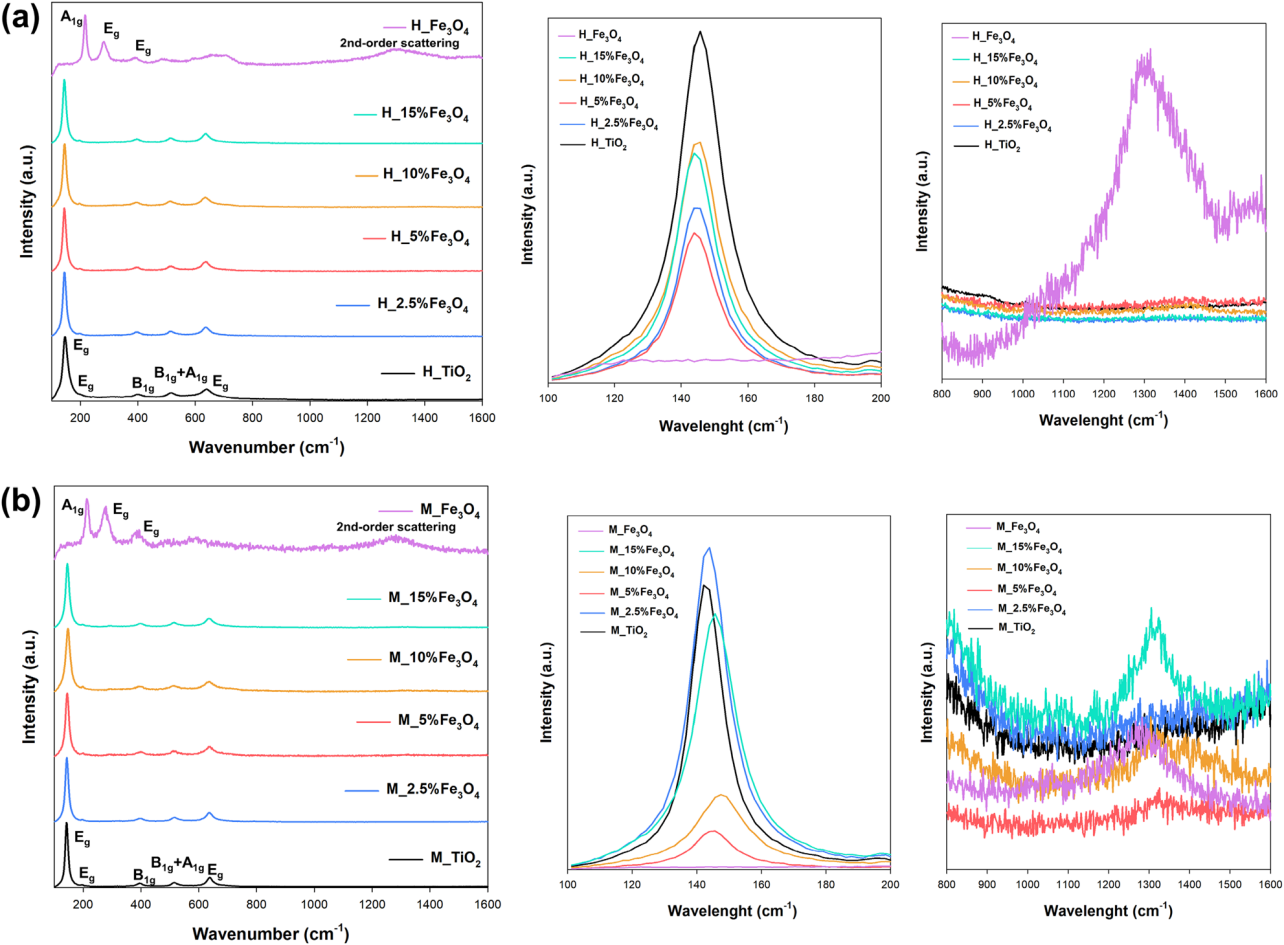
The Raman spectra for TiO_2_–Fe_3_O_4_ systems synthesized by (**a**) hydrothermal and (**b**) microwave methods.

**Figure 3:**
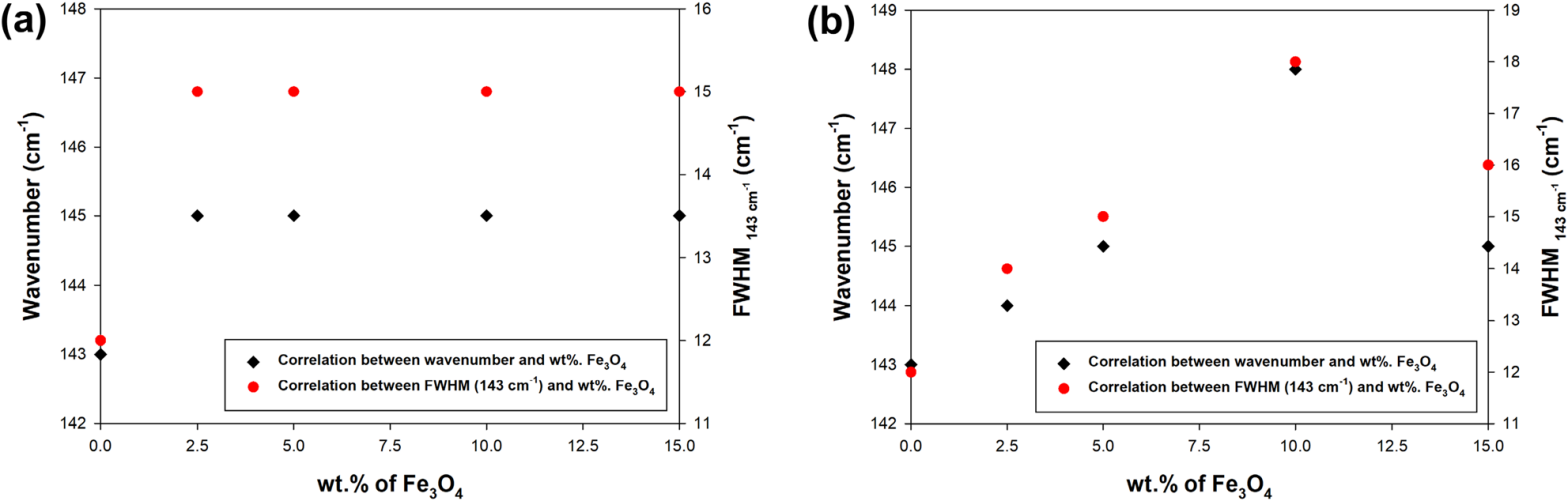
Raman spectra in the wavenumber range of 100–200 cm^−1^ for TiO_2_–Fe_3_O_4_ systems synthesized using (**a**) conventional and (**b**) microwave treatment, in particular the position of the anatase band E_g_ (143 cm^−1^) and FWHM E_g_ (143 cm^−1^) depending on wt% Fe_3_O_4_.

Raman spectroscopy was used to analyze TiO_2_–Fe_3_O_4_ systems, and the collected spectra are presented in Fig. 2. In the spectra for the reference samples H_TiO_2_ and M_TiO_2_, five characteristic bands were observed at 140 cm^−1^ (E_g_), 195 cm^−1^ (E_g_), 395 cm^−1^ (B_1g_), 520 cm^−1^ (B_1g_ + A_1g_), and 640 cm^−1^ (E_g_)^43^, corresponding to anatase. For samples H_Fe_3_O_4_ and M_Fe_3_O_4_, bands at 210 cm^−1^ (A_1g_), 271 cm^−1^ (E_g_), and 385 cm^−1^ (E_g_), as well as the band responsible for the 2nd-order scattering, confirming the magnetite structure^44^, were observed. By analyzing the Raman spectra for the TiO_2_–Fe_3_O_4_ systems, clear differences were observed between the results obtained for conventional and microwave treatments. For the samples of the H_TiO_2_–Fe_3_O_4_ series, peaks of the anatase structure were detected, while for magnetite structure, only a weak peak from 2nd-order scattering was observed. The absence of additional bands from magnetite may be due to the high intensity of bands characteristic of TiO_2_. In the case of the M_TiO_2_–Fe_3_O_4_ series, strong symmetry bands E_g_, B_1g_, and B_1g_ + A_1g_ were found, indicating the anatase crystal structure. Additionally, as in the case of materials obtained using conventional processing, bands from 2nd-order scattering were noted. However, attention should be paid to the higher intensity of the mentioned band for the M_TiO_2_–Fe_3_O_4_ series. Furthermore, it should be noted that for materials synthesized using the microwave treatment, a shift of the peak towards higher wavenumbers is observed. Therefore, the location of the E_g_ band (143 cm^−1^) and its full width at half maximum (FWHM) were analyzed depending on the Fe_3_O_4_ weight ratio, and the obtained data are presented in Fig. 3.

Based on the analyzed Raman spectra in the wavenumber range of 100–200 cm^−1^, it was confirmed that for TiO_2_–Fe_3_O_4_ materials fabricated using conventional processing, no significant changes in the position of the anatase E_g_ peak (143 cm^−1^) were noted. This confirms previous observations that anatase and magnetite coexist as two separate phases in the resulting systems. On the other hand, for materials obtained using the microwave process, a shift of the E_g_ band (143 cm^−1^) and its half-width towards higher values of the wavenumber was observed. It is worth noting that this relationship is linear up to the limit value of 10 wt% Fe_3_O_4_. Raman spectroscopy is highly sensitive to changes in molecules and is often used as a probe to determine the local structural properties of nanopowders^12,45^. Hardwick et al.^46^ have reported that the insertion of Li ions into anatase-type nanocrystalline TiO_2_ deforms the anatase tetragonal lattice, transforming it into an orthorhombic structure that creates new Raman active modes. Palomino-Merino et al.^47^ reported that in Er-doped TiO_2_ material, erbium induces a slight structural deformation that causes the origin of an anatase peak E_g_ (143 cm^−1^) to redshift. The main reason for the mentioned shift is the insertion of the Er ions into the TiO_2_ lattice. Based on the analysis of the Raman spectra, it can be inferred that the observed red shift in the M_TiO_2_–Fe_3_O_4_ sample series is likely a result of the incorporation of Fe ions into the TiO_2_ lattice. The effective incorporation of iron into the titanium dioxide lattice appears to occur up to a content of 10 wt% Fe_3_O_4_. However, for higher contents of iron (II, III) oxide, a blue shift in the Raman spectrum is observed. This could be due to a reduced number of sites in the TiO_2_ lattice where iron can be incorporated, resulting from a decrease in its concentration.

### Morphology

**Figure 4:**
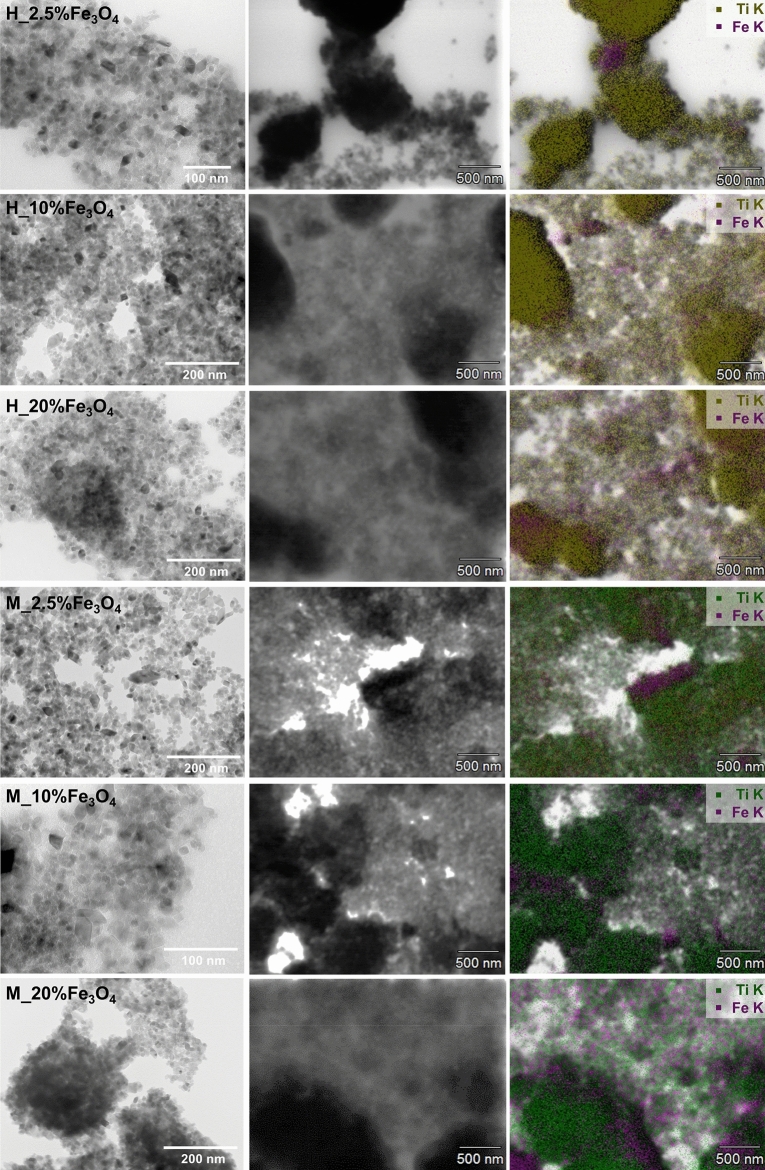
From left: TEM, STEM images, and EDX maps for TiO_2_–Fe_3_O_4_ systems synthesized using conventional and microwave treatment.

TEM and EDX analyses were carried out to characterize the morphology of the obtained materials in terms of particle shape and size. The images obtained in Fig. 4 reveal that nanoparticles of varying shapes, such as spherical, cubic, and octahedral, were observed in all the analyzed materials. The dimensions of the nanoparticles were dependent on the treatment method used. For instance, the conventional processing method resulted in particles with an average size of 25–50 nm, while the microwave method produced smaller nanoparticles with an average size of 10–20 nm. It is worth noting that the obtained materials exhibit a high tendency to aggregate nanoparticles regardless of the treatment method used, which is confirmed by the BET results (refer to Table S3 in the Supplementary Materials). This observation is consistent with previous studies, such as Belousov et al.^48^, who reported that microwave radiation could be used to not only expedite the synthesis of particles but also obtain more uniform materials compared to conventional heating.

EDX elemental mapping shown in Fig. 4 (The table containing information on the elemental composition is included in the Supplementary Materials—Table S4) indicates that the distribution of titanium and iron in the H_TiO_2_–Fe_3_O_4_ series is not uniform, with a high aggregation of nanoparticles into larger structures with a diameter of approximately 500 nm. Iron is primarily located in these nanoparticle aggregates. The aggregation process may be attributed to the long conventional treatment time, which is based on the collision of molecules and heat conduction mechanisms. In turn, the TiO_2_–Fe_3_O_4_ systems prepared using the microwave method exhibited a more homogeneous distribution of elements (except for sample M_2.5%Fe_3_O_4_) compared to the previously described samples. Additionally, as the content of Fe_3_O_4_ in the M_TiO_2_–Fe_3_O_4_ series increased gradually to 20 wt%, the presence of larger aggregates could be observed.

### Magnetic measurement

**Figure 5:**
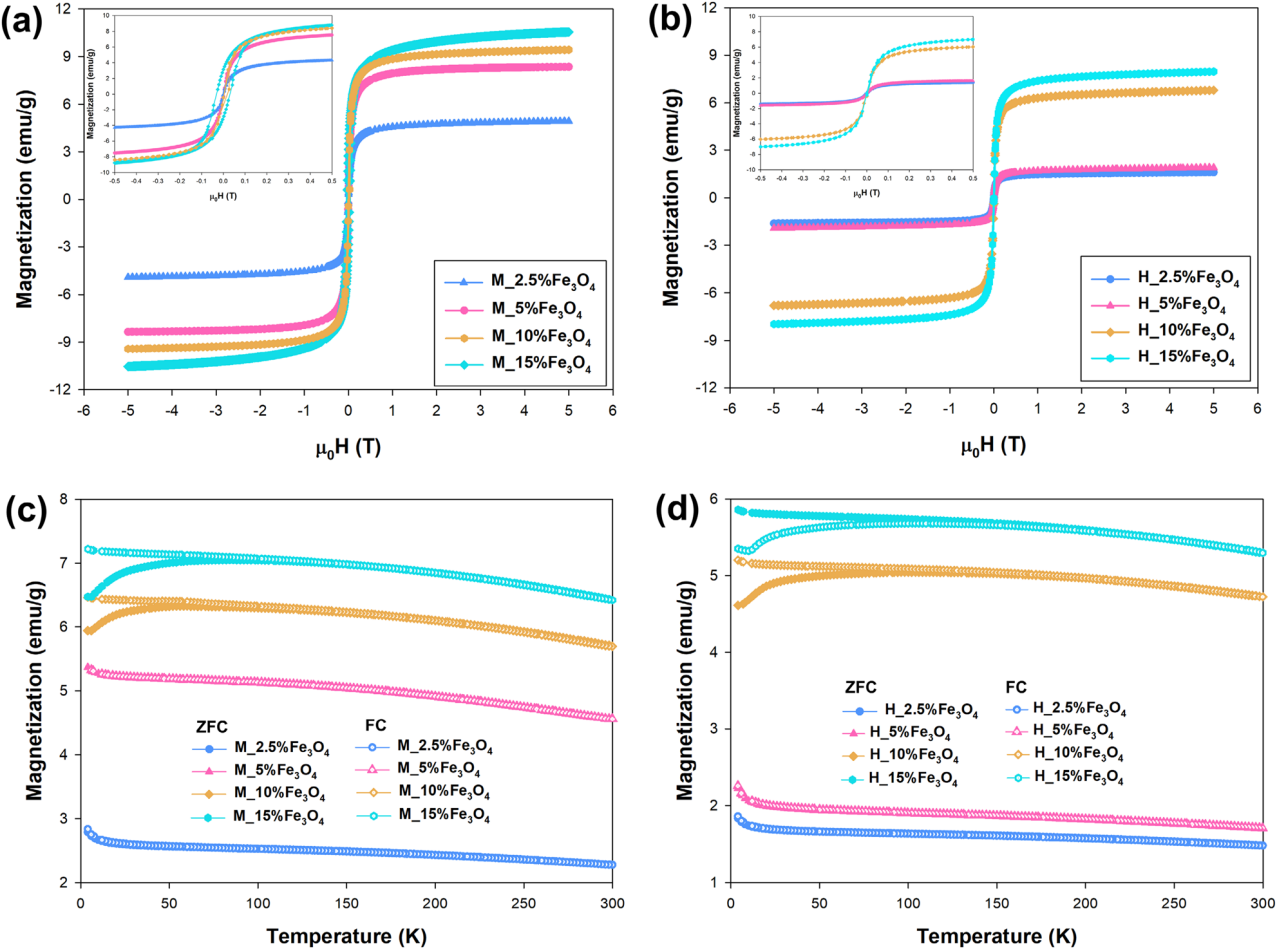
Magnetic properties of TiO_2_–Fe_3_O_4_ systems synthesized using (**a**,**c**) microwave and (**b**,**d**) conventional treatment: (**a**,**b**) dependence of magnetization on temperature for all tested samples measured in a constant magnetic field μ_0_H = 0.1 T with zoom-in of the low-field region, and (**c**,**d**) magnetization as a function of external magnetic field for oxide systems measured at T = 300 K.

All results of magnetic measurements of synthesized TiO_2_–Fe_3_O_4_ systems are collected in Fig. 5. As inferred, the TiO_2_–Fe_3_O_4_ systems show similar magnetic properties as pure nano-Fe_3_O_4_. The temperature dependence of magnetization (Fig. 4c,d) shows a strong bifurcation of the Zero-Field-Cooling (ZFC) curve and Field-Cooled (FC) curve. For all the analyzed materials, no Verwey transition was observed, which is related to the small size of the magnetite nanoparticles^49^. On the other hand, for the curve measured during cooling in zero magnetic fields (ZFC), a clear broad maximum (~ 115 K) is seen, which is related to the blocking temperature (TB) of the magnetic nanoparticles^50^. The samples containing 10 and 15 wt% Fe_3_O_4_ show a clear broad maximum for the ZFC curve (at ~ 50 K and ~ 90 K, respectively), which is invisible for samples with low magnetite content (2.5% and 5 wt% Fe_3_O_4_). For the above-mentioned materials, no splitting of the ZFC and FC curves was observed either. This indicates a different size of magnetite nanoparticles in the analyzed materials—which is consistent with the previously presented XRD analysis^51^. In the case of magnetization curves (M(H)) measured at 300 K, we do not observe large differences between samples with different percentages of Fe_3_O_4_. Regardless of the analyzed material, no remanence or magnetic hysteresis was observed. It should be noted that the materials obtained using microwave treatment have higher magnetization values compared to the products of conventional processing. This is mainly due to the differences in the size of the crystallites and the content of the magnetite phase in the analyzed materials. Nevertheless, the obtained results suggest superparamagnetic properties for all obtained TiO_2_–Fe_3_O_4_ systems synthesized using the conventional and microwave pathways.

### Optical properties

**Figure 6:**
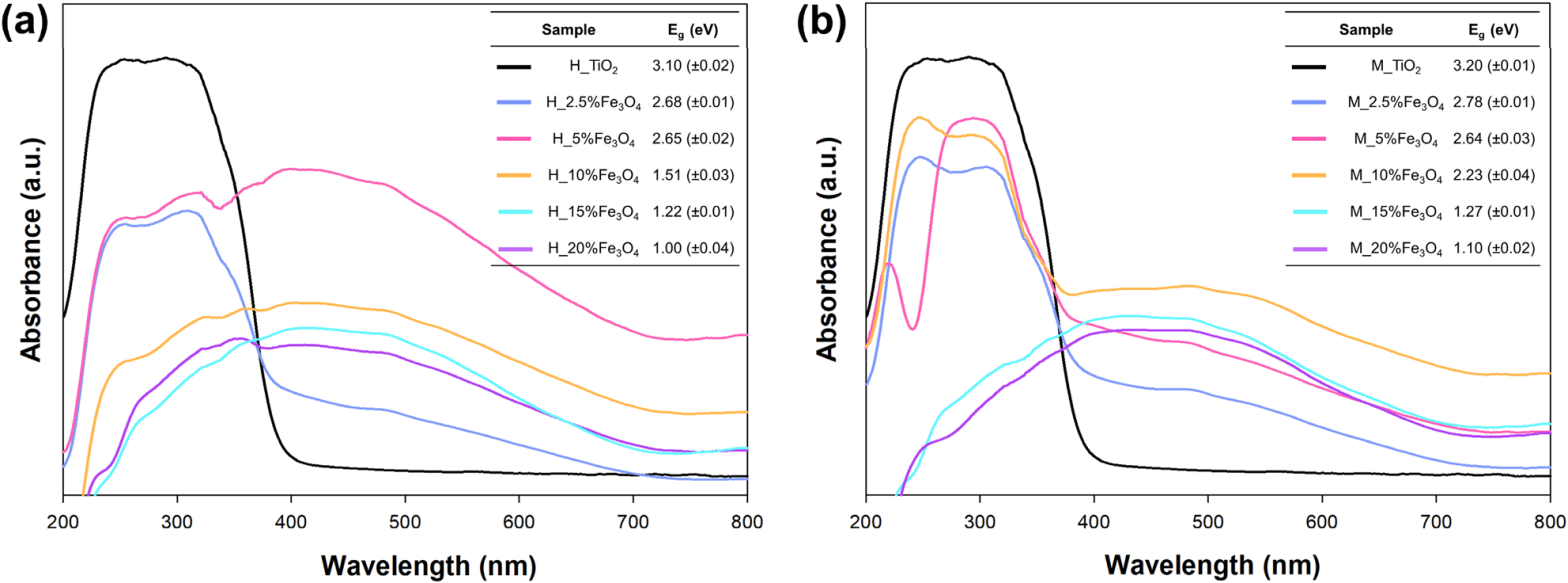
The DRS spectra for TiO_2_–Fe_3_O_4_ systems synthesized by (**a**) conventional hydrothermal and (**b**) microwave methods.

Diffuse reflectance spectroscopy (DRS) was applied to evaluate the optical response of the synthesized materials. The UV–vis diffuse reflectance spectra of TiO_2_–Fe_3_O_4_ composites and the reference TiO_2_ samples are presented in Fig. 6. Both reference materials (H_TiO_2_ and M_TiO_2_) exhibited a distinct absorption edge around 385 nm, characteristic of anatase^52,53^. For all TiO_2_–Fe_3_O_4_ composites, regardless of the synthesis route, an extension of light absorption toward the visible region was observed. This red shift can be attributed to interfacial charge-transfer interactions between Fe_3_O_4_ and TiO_2_^54^, which generate additional electronic states near the band edges and enhance visible-light absorption. The presence of iron oxide nanoparticles may also affect the local electronic environment of TiO_2_, leading to small modifications in the energy separation of its band edges and the quantization of energy levels^55,56^. As a result, the composites display improved utilization of photons emitted by the applied LED source. The apparent optical band gap values, estimated from Tauc plots of (F(R)·E)^0.5^ versus photon energy E, are provided in the Supplementary Materials (Fig. S2). These values should be regarded as comparative indicators of optical threshold shifts rather than as intrinsic band gaps, since the Tauc approach is most reliable for homogeneous semiconductors.

Samples synthesized via conventional hydrothermal processing exhibited slightly lower apparent optical thresholds, likely due to enhanced light scattering within aggregated particles. Conversely, microwave-assisted synthesis produced smaller, more uniformly distributed nanoparticles, which favored a more efficient light-harvesting response. The red-shifted absorption and Raman peak displacement observed for the microwave-derived materials indicate stronger interfacial coupling between TiO_2_ and Fe_3_O_4_ domains. These results collectively suggest that microwave processing promotes intimate TiO_2_–Fe_3_O_4_ interfacial contact, improving the utilization of near-UV photons under the employed LED irradiation.

### Photocatalytic tests

**Figure 7:**
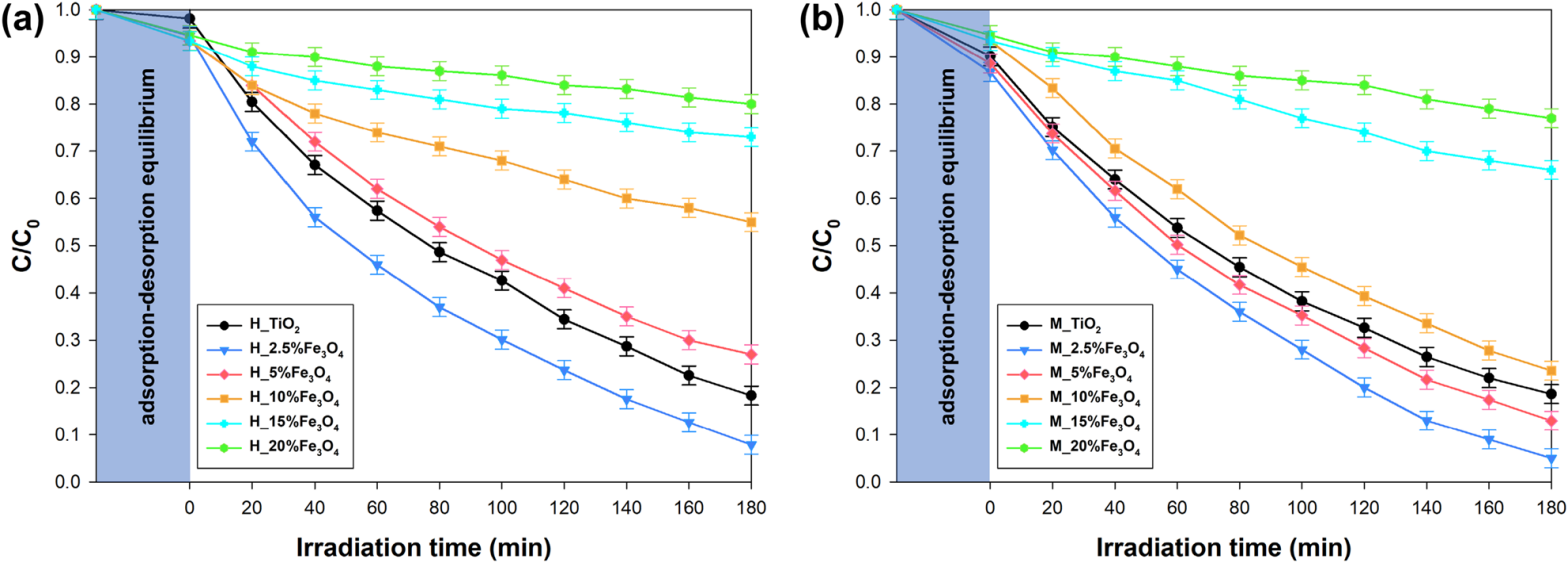
The results of MNZ photodegradation using TiO_2_–Fe_3_O_4_ systems synthesized by (**a**) conventional hydrothermal and (**b**) microwave methods.

Photocatalytic degradation of metronidazole (20 mg/L) was carried out using a dosage of photocatalysts (1 g/L) and the results are shown in Fig. 7. The amount of the photocatalyst used were selected based on other scientific reports related to the photo-oxidation of metronidazole. Neghi et al.^57^ published a study using a dose of 2.5 g/L TiO_2_ which resulted in the full degradation of metronidazole (0.1 mg/L). In another report, Neghi et al.^58^ applied a lower photocatalyst dosage (0.3 g/L) for the degradation of 10 mg/L MNZ solution and observed 18% degradation after 120 min indicating lower dosage was not effective for the removal of metronidazole for a short period experiment.

Figure 7a shows the photooxidation efficiency of metronidazole using TiO_2_–Fe_3_O_4_ photocatalysts synthesized via conventional hydrothermal treatment. Based on the results, the adsorption of MNZ on the surface of TiO_2_–Fe_3_O_4_ systems was in the range of 6–8% after 30 min. The photocatalytic degradation using mentioned systems showed better results only for the H_2.5%Fe_3_O_4_. Based on the findings, the removal efficiency of MNZ was near 90%, while for the reference H_TiO_2_ was approx. 80%. The further increase in the magnetite add-on resulted in a significant decrease in the efficiency of the photocatalytic activity. For the H_20% Fe_3_O_4_ sample, only 20% of the MNZ removal efficiency was obtained. The main reason for the decrease in photodegradation efficiency is the previously described change in the band gap energy. The increase in the content of Fe_3_O_4_ in the case of materials obtained using conventional processing was associated with the deterioration of the optical properties of these materials. In addition, the morphology, i.e. aggregates of nanoparticles with a size of nearly 500 nm, may promote the higher recombination rate of charge carriers^59^. On the other hand, photocatalytic degradation catalyzed by TiO_2_–Fe_3_O_4_ fabricated by microwave process (Fig. 7b) showed better results for samples containing 2.5% and 5 wt% Fe_3_O_4_. The removal efficiency of MNZ was 96% (M_2.5%Fe_3_O_4_) and 85% (M_5%Fe_3_O_4_), that significantly was higher than the removal efficiencies of M_TiO_2_ (80%) after 180 min of UV-LED irradiation.

Photoluminescence spectroscopy was utilized to comprehensively analyze the photocatalytic performance of the synthesized TiO_2_–Fe_3_O_4_ materials (see Fig. S3 in Supplementary Materials). The results showed a luminescence band close to ~ 450 nm, which is characteristic of bare TiO_2_. However, in the case of materials containing 2.5 and 5 wt% Fe_3_O_4_, there was a quenching of the luminescence, indicating suppression of the recombination of photogenerated holes and electrons in the TiO_2_–Fe_3_O_4_ systems. This can be attributed to the fact that excited electrons from the valence band of titanium dioxide pass to the conduction band and subsequently to the Fe_3_O_4_ structure. The increase in Fe_3_O_4_ content also caused quenching of the luminescence compared to bare TiO_2_, but an increase compared to materials containing lower Fe_3_O_4_ content. This may be due to the shielding effect of Fe_3_O_4_, as mentioned in previous studies by Cheng et al.^60^ and Stefan et al.^61^. However, a high content of Fe_3_O_4_ can act as an electron trap, leading to a reduction in the charge transfer between TiO_2_ and the photocatalytically active substrate, thereby limiting the photocatalytic performance of TiO_2_. The lower luminescence of materials obtained in the microwave process may indicate a greater ordering of the structure and the absence of recombination centers. Nevertheless, in both analyzed cases, the formation of TiO_2_–Fe_3_O_4_ systems leads to the quenching of luminescence, which has a key impact on the improvement of photocatalytic activity in the metronidazole removal process.

In order to gain a deeper understanding of the degradation mechanism in the presence of TiO_2_–Fe_3_O_4_ photocatalysts, we introduced scavengers capable of capturing photogenerated electrons, holes, and primary reactive oxygen species, such as hydroxyl radicals (^*^OH) and superoxide radical anions (^*^O_2_^−^), into the metronidazole solution. The obtained results were collected and presented in Fig. S4 in Supplementary Materials. The introduction of ammonium oxalate and silver nitrate as scavengers for electrons and holes resulted in a slight decrease of approximately 10% in the photooxidation efficiency of metronidazole (MNZ). This suggests that the recombination of charge carriers (electrons and holes) does not have a significant impact on the overall photo-oxidation efficiency, as confirmed by the analysis of photoluminescence spectra. On the other hand, the addition of tert-butanol as a scavenger for hydroxyl radicals (^*^OH) had a significant effect, reducing the photodegradation efficiency by approximately 50% for the analyzed materials. This indicates that ^*^OH is an active species involved in the photooxidation process. Furthermore, the scavenging of superoxide radical anions (^*^O_2_^−^) had a considerable impact on the efficiency of MNZ removal, resulting in a decrease of approximately 60%. This highlights the crucial role played by ^*^O_2_^−^ in the photocatalytic degradation mechanism. Based on these observations, it can be inferred that the mechanisms occurring in aquatic environments are primarily governed by the generation of reactive oxygen species (ROS), including hydroxyl radicals and superoxide radical anions.

**Figure 8:**
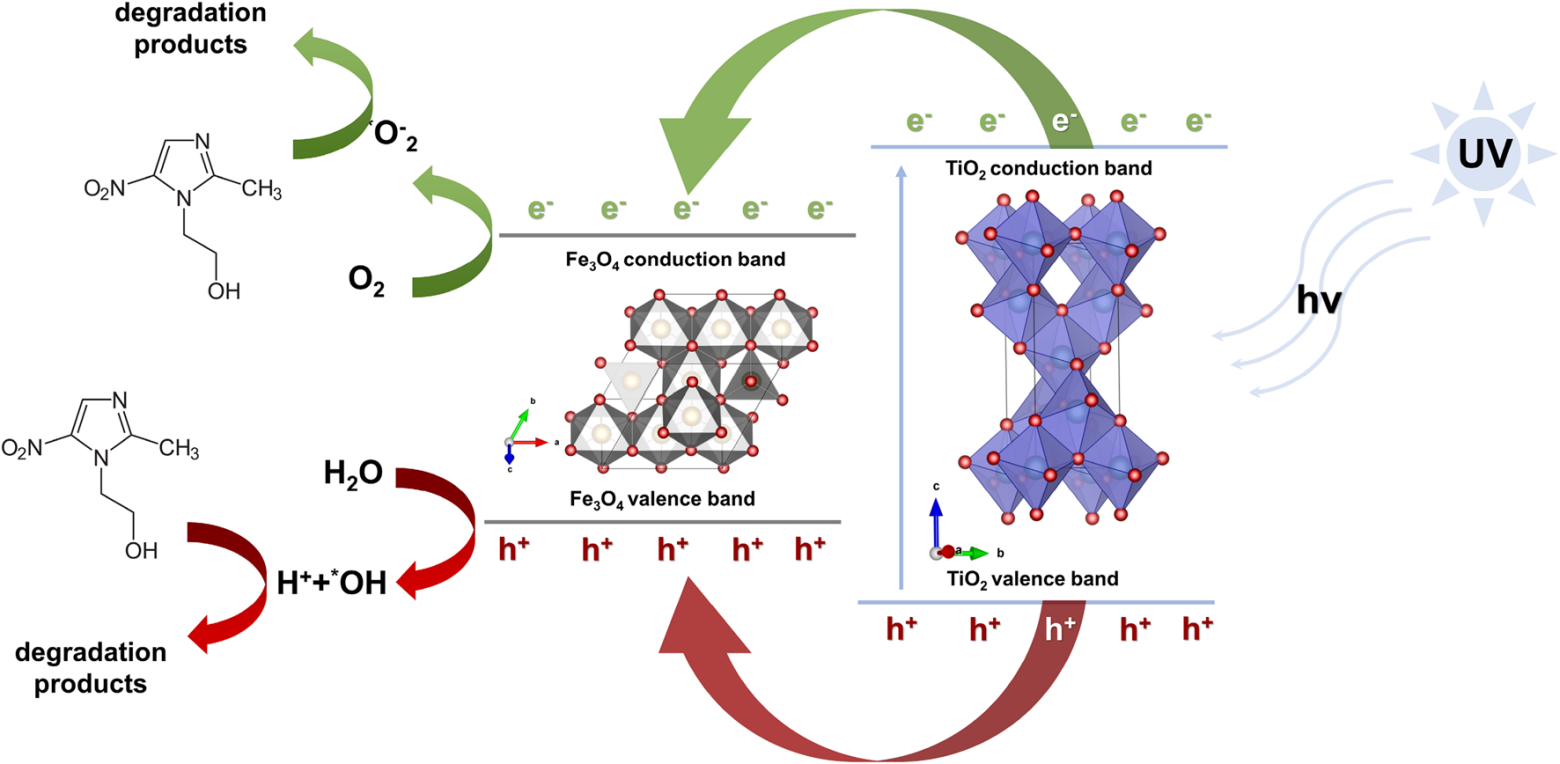
Proposed mechanism of photooxidation of metronidazole using TiO_2_–Fe_3_O_4_ photocatalysts.

Another factor determining the improvement of MNZ removal efficiency in the case of materials obtained by the microwave method is the possibility of creating a heterojunction between the system components—TiO_2_ and Fe_3_O_4_^62,63^. Based on the previously described Raman data, it was indicated that the possibility of introduction of Fe ions into the TiO_2_ lattice during microwave processing. In addition, these materials are characterized by smaller particle sizes and do not tend to agglomerate, which may improve optical properties by not trapping TiO_2_ in Fe_3_O_4_ aggregates. On this basis, a mechanism based on type I heterojunction was proposed^64^. The Fe_3_O_4_ conduction band is below the TiO_2_ conduction band, while the Fe_3_O_4_ valence band is above the TiO_2_ valence band. Because electrons and holes gain energy by moving down and up, respectively. Photoexcited electrons can transfer from the conduction band of TiO_2_ to the conduction band of Fe_3_O_4_, while holes can be transferred from the valence band of TiO_2_ to the valence band of Fe_3_O_4_ when there is sufficient contact between both semiconductors. In this way, all charge carriers accumulate on Fe_3_O_4_. The disadvantage of the proposed mechanism is that both electrons and holes accumulate on the same semiconductor, thus electron–hole pairs cannot be separated efficiently^65^. However, many scientific reports report that the type I heterojunction makes it possible to improve the photoactivity of the final material. Among others, Monitz et al.^66^ indicated that the TiO_2_(P25)–CuO material may be characterized by the higher efficiency of photocatalytic processes compared to the reference material. Figure 8 illustrates the proposed mechanism, which is consistent with the type I heterojunction.

However, in the case of the synthesized TiO_2_–Fe_3_O_4_ systems, the improvement in photoactivity can be attributed to the combined effects of interfacial charge transfer and an apparent narrowing of the optical band gap, which together enable a higher utilization of radiation from the applied LED light source. Moreover, Chu et al.^11^ reported that the anatase phase of TiO_2_ in TiO_2_–Fe_3_O_4_ systems exhibits characteristics consistent with a type I heterojunction mechanism. In this configuration, the anatase surface can efficiently generate hydroxyl radicals that react with pollutants and enhance photocatalytic degradation. The presence of iron species near the TiO_2_–Fe_3_O_4_ interface may promote the formation of lattice vacancies, thereby increasing the adsorption of surface water and facilitating the generation of hydroxyl radicals^67^. Iron ions, particularly Fe^3+^, can act as acceptors of photogenerated charge from the valence band of anatase, mitigating recombination by transiently capturing photo-induced electrons. Fe^2+^ ions, having one more electron than the half-filled 3d configuration, can easily donate this excess electron to surface-adsorbed oxygen, leading to the formation of superoxide anions^10^. Additionally, Fe^3+^ ions can accept photogenerated holes (h+) and oxidize to Fe^4+^, which may further react with surface hydroxide ions to produce hydroxyl radicals or recombine with trapped electrons^68,69^. Overall, the superior photocatalytic performance of the TiO_2_–Fe_3_O_4_ systems can be ascribed to the synergistic action of the type I heterojunction and the dynamic Fe^3+^/Fe^2+^ redox cycling at the interface, which collectively enhance charge separation and reactive oxygen species formation while reducing electron–hole recombination.

Thermogravimetric analysis (TGA) was conducted to confirm the exceptional stability of the synthesized materials (refer to Fig. S5 in the Supplementary Materials). TGA analysis was performed on the most efficient photocatalysts, and the results showed that the TiO_2_–Fe_3_O_4_ catalysts demonstrated high thermal resistance regardless of the synthesis method utilized (conventional hydrothermal or microwave process). The materials exhibited a mass loss of just over 3% up to 1000 °C.

The H_2.5%Fe_3_O_4_ and M_2.5%Fe_3_O_4_ photocatalysts were selected for the reusability test. The five subsequent cycles of MZN degradation were performed to study the photocatalytic reusability, as presented in Fig. S6 in Supplementary Materials. At each run’s end, the TiO_2_–Fe_3_O_4_ photocatalyst was separated using an external magnetic field. At the end of each cycle, the photocatalyst was removed from the degraded solution and transferred to the next photoreactor. The photocatalyst was not subjected to any additional treatment between catalytic cycles. The efficiency of photocatalytic degradation was maintained without a significant decline, even in the 5th cycle.

The photocatalytic system must be able to remove contaminants efficiently. However, nowadays, the economy of this process, and in particular its energy consumption, is extremely important. Therefore, taking into account the available scientific reports for the photocatalysts H_2.5%Fe_3_O_4_ and M_2.5%Fe_3_O_4_, the electrical energy per order (E_EO_)^70^ parameter was determined. It is the energy required for achieving the chosen level of abatement, for a fixed volume of wastewater, which can be thus calculated through the following formula (Eq. 3). Values of E_E0_ not higher than 2.5 are considered suitable for practical applications.

3 $$E_{EO} = \frac{P \cdot t \cdot 1000}{{V \cdot 60 \cdot {\mathrm{log}}\left( {\frac{{C_{0} }}{{C_{t} }}} \right)}}$$

where *P* is the lamp power (kW), _k_ is the pseudo-first-order constant (min^−1^), *V is* the volume of the reactor, and *t* is irradiation time. From the E_EO_ values, it was evident that the photocatalytic system with TiO_2_–Fe_3_O_4_ photocatalyst and UV-LED light source was highly energy efficient. For analyzed samples, the mentioned parameter was 0.387 kWh m^−3^ order^−1^ for H_2.5%Fe_3_O_4_ and 0.308 kWh m^−3^ order^−1^ for M_2.5%Fe_3_O_4_. When comparing these values with similar photocatalytic systems used for metronidazole degradation, it should be noted that these values are lower than those reported by Pan et al.^71^. This fact confirms that tailor-made LED photocatalytic systems may in the future be used as part of wastewater treatment technology.

## Conclusions

This study aimed to compare the conventional and microwave treatment methods for synthesizing TiO_2_–Fe_3_O_4_ photocatalyst used for metronidazole decomposition. The XRD results suggested that the reduction in crystallite size of Fe-doped materials compared to undoped nanoparticles may be related to the transfer of a small amount of Fe ions to the interstitial or TiO_2_ substitution site. The Raman spectra analysis revealed a shift of E_g_ band (143 cm^−1^) towards higher wavenumbers in the materials synthesized using the microwave process. This shift is likely the result of the incorporation of Fe ions into the TiO_2_ lattice. All synthesized systems show superparamagnetic properties and no Verwey transition or magnetic hysteresis was observed. However, the materials obtained using microwave treatment have higher magnetization values than those obtained using conventional processing. The DRS spectra showed a red shift of the absorption band edge and lower bandgap energy in all systems, which is related to the charge transfer transition between the electrons of the Fe_3_O_4_ nanoparticles and TiO_2_. The enhanced photoactivity is attributed to the possibility of creating a heterojunction between TiO_2_ and Fe_3_O_4_, leading to the accumulation of all charge carriers on Fe_3_O_4_. This approach enables the production of tailor-made photocatalytic systems where the photocatalyst and light source are spectrally matched.

## Supplementary Information

Supplementary Information.

## Data Availability

The data that support the findings of this research are available from the corresponding author upon reasonable request.
