# Correction: Comparative study of TiO_2_–Fe_3_O_4_ photocatalysts synthesized by conventional and microwave methods for metronidazole removal

**DOI:** 10.1038/s41598-026-37543-6

**Published:** 2026-02-18

**Authors:** Adam Kubiak

**Affiliations:** Faculty of Chemistry, Adam Mickiewicz University, Uniwersytetu Poznanskiego 8, 61614 Poznan, Poland

Correction to: *Scientific Reports* 10.1038/s41598-023-39342-9, published online 26 July 2023

The original version of this Article contained errors in the Results and discussions under the subheadings ‘Optical properties’ and ‘Photocatalytic tests’ where the original phrasing may unintentionally suggest intrinsic band-gap narrowing of TiO2, whereas the experimental data support an apparent optical threshold shift associated with interfacial charge-transfer effects in the TiO2–Fe3O4 system.

The original Article has been corrected.

