# Supplementary material for: Comparative study of TiO2–Fe3O4 photocatalysts synthesized by conventional and microwave methods for metronidazole removal
Source: Sci Rep. 2023 Jul 26;13:12075. doi: 10.1038/s41598-023-39342-9 (PMC10371990; doi:10.1038/s41598-023-39342-9)
Supplement: Supplementary file 1 — Supplementary Information. [file 41598_2023_39342_MOESM1_ESM.pdf]

Comparative study of TiO_2-Fe_3O_4 photocatalysts synthesized by conventional and microwave methods for metronidazole removal

Adam Kubiak*

Adam Mickiewicz University, Poznan, Faculty of Chemistry, Uniwersytetu Poznanskiego 8, PL-61614 Poznan, Poland

**Corresponding author: adam.kubiak@amu.edu.pl; Tel.: +48 61 829 17 21*

1. Experimental section

1.1. Synthesis of TiO_2-Fe_3O_4 systems

Table S1. Determination of samples of oxide systems and the amount of substrates used

Sample	Amount of Fe^{2+}:Fe^{3+} (g)	Amount of TiO_2 (g)	wt.% of Fe_3O_4
**H or M_TiO_2**		-	0
**H or M_2,5%Fe_3O_4**		3.5	2.5
**H or M_5%Fe_3O_4**		1.75	5
**H or M_10%Fe_3O_4**	0.1 : 0.2	0.87	10
**H or M_15%Fe_3O_4**		0.58	15
**H or M_20%Fe_3O_4**		0.44	20
**H or M_Fe_3O_4**		-	100

1.2. Characterization of the LED light source

The degradation of metronidazole, used as a model pharmaceutical, was assessed to determine the photooxidation activity of the synthesized systems. A novel LED lamp utilizing the COB (chip-on-board) system was employed as the light source. For this study, an LED system was utilized that is based on a diode with a wavelength of 395-405 nm and a power of 20W (BRIDGELUX, USA). The diode has an aluminum passive radiator to dissipate the generated heat due to the high power used. Finally, the resulting system was connected to the driver

(TOPXIN Electronics Co., Shenzhen, China). Measurement of the power and energy consumption of the UV-LED lamp was carried out using a GB202 wattmeter (GreenBlue, China). Figure S1 shows the resulting UV-LED light source.

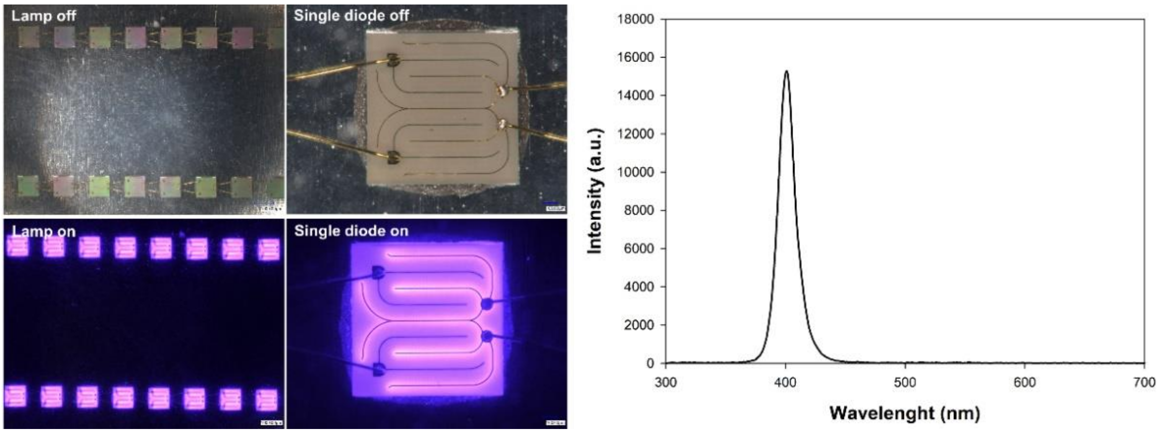

Fig. S1. The photo of the lamp off/on, single diode off/on, and spectrum of the used COB LED.

2. Results

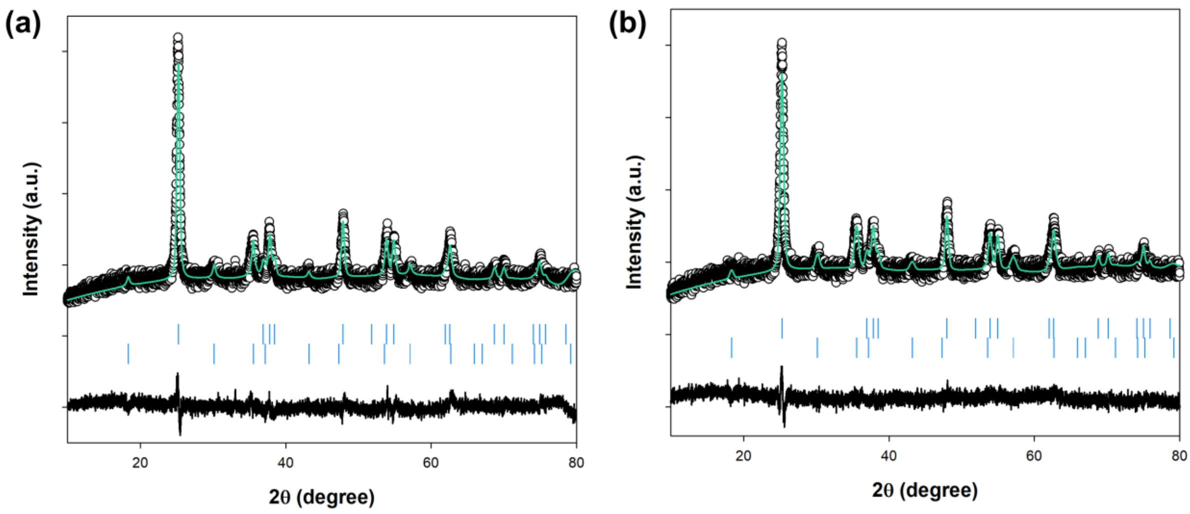

Fig. S1. Example of Rietveld refinement for (a) H_10%Fe_3O_4, and (b) M_10%Fe_3O_4.

Table S2. The phase composition, average crystalline size, and lattice parameters for TiO_2-Fe_3O_4 systems were obtained from XRD.

Sample	Phase composition (%)		The average crystallite size (nm)		Lattice parameters		
					anatase		magnetite
	anatase	magnetite	anatase	magnetite	*a* (Å)	*c* (Å)	*c* (Å)
**conventional treatment**							
**H_2.5%Fe_3O_4**	97 (±0.2)	3 (±0.5)	18.4 (±0.1)	14.2 (±0.1)	3.78267	9.49916	8.37069
**H_5%Fe_3O_4**	94 (±0.3)	6 (±1.3)	18.4 (±0.1)	13.1 (±0.3)	3.78872	9.49916	8.36364
**H_10%Fe_3O_4**	90 (±0.4)	10 (±1.0)	18.7 (±0.2)	13.3 (±0.5)	3.79251	9.50103	8.35607
**H_15%Fe_3O_4**	83 (±0.4)	17 (±1.0)	18.5 (±0.2)	11.1 (±0.6)	3.79102	9.50384	8.35607
**H_20%Fe_3O_4**	80 (±0.2)	20 (±0.4)	18.6 (±0.1)	11.5 (±0.4)	3.79267	9.50395	8.35810
**microwave treatment**							
**M_2.5%Fe_3O_4**	97 (±0.3)	3 (±0.4)	18.0 (±0.1)	11.8 (±1.1)	3.80184	9.48145	8.39004
**M_5%Fe_3O_4**	91 (±0.2)	9 (±1.0)	18.0 (±0.1)	11.2 (±0.7)	3.80061	9.48232	8.38153
**M_10%Fe_3O_4**	88 (±0.6)	12 (±0.5)	17.8 (±0.1)	11.0 (±0.4)	3.79388	9.48561	8.37461
**M_15%Fe_3O_4**	85 (±0.7)	15 (±1.0)	17.8 (±0.1)	10.3 (±0.3)	3.79099	9.48136	8.35246
**M_20%Fe_3O_4**	76 (±0.4)	24 (±0.9)	17.3 (±0.1)	9.0 (±0.2)	3.78991	9.49232	8.36002

Table S3. Parameters of the porous structure for the obtained TiO_2-Fe_3O_4 materials.

Sample	A_{BET} (m^2/g)	V_p (cm^3/g)	S_p (nm)
**conventional treatment**			
H_TiO_2	107	0.335	21
H_2.5%Fe_3O_4	100	0.317	18
H_5%Fe_3O_4	99	0.304	17
H_10%Fe_3O_4	90	0.300	15
H_20%Fe_3O_4	82	0.297	12
**conventional treatment**			
M_TiO_2	120	0.387	27
M_2.5%Fe_3O_4	115	0.358	26
M_5%Fe_3O_4	111	0.331	26
M_10%Fe_3O_4	106	0.330	24
M_20%Fe_3O_4	101	0.328	22

Table S4. Results of EDS analysis for TiO_2-Fe_3O_4 oxide systems synthesized by conventional hydrothermal and microwave methods

Sample	Element	Mass percentage (%)
**conventional method**		
**H_2.5%Fe_3O_4**	Ti	49.7
	O	46.6
	Fe	3.7
**H_10%Fe_3O_4**	Ti	48.1
	O	44.3
	Fe	7.6
**H_20%Fe_3O_4**	Ti	47.0
	O	37.6
	Fe	15.4
**microwave method**		
**M_2.5%Fe_3O_4**	Ti	50.9
	O	46.6
	Fe	2.5
**M_10%Fe_3O_4**	Ti	44.9
	O	42.1
	Fe	13.1
**M_20%Fe_3O_4**	Ti	42.2
	O	42.0
	Fe	15.7

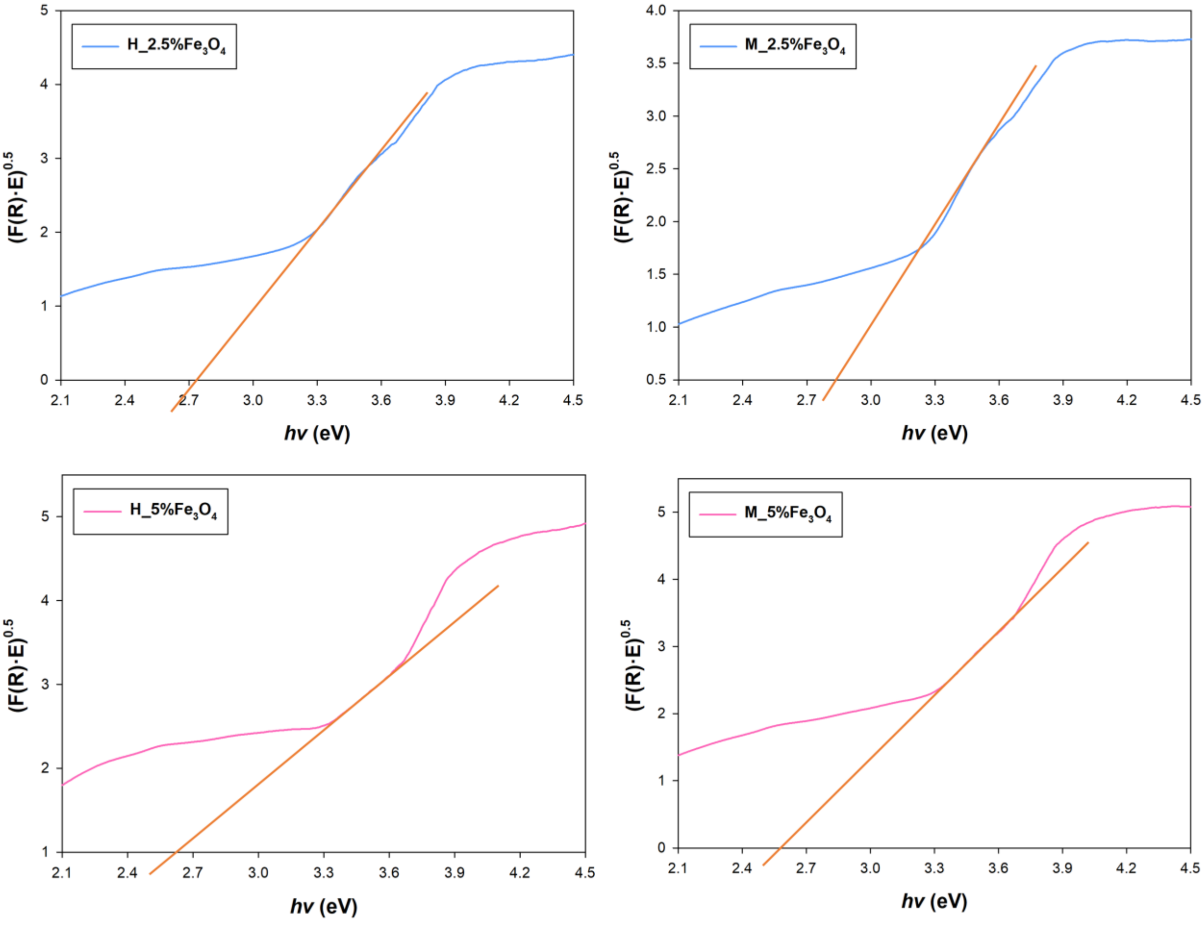

Fig. S2. The Tauc plot to determine the band gap energy for selected TiO_2-Fe_3O_4 systems.

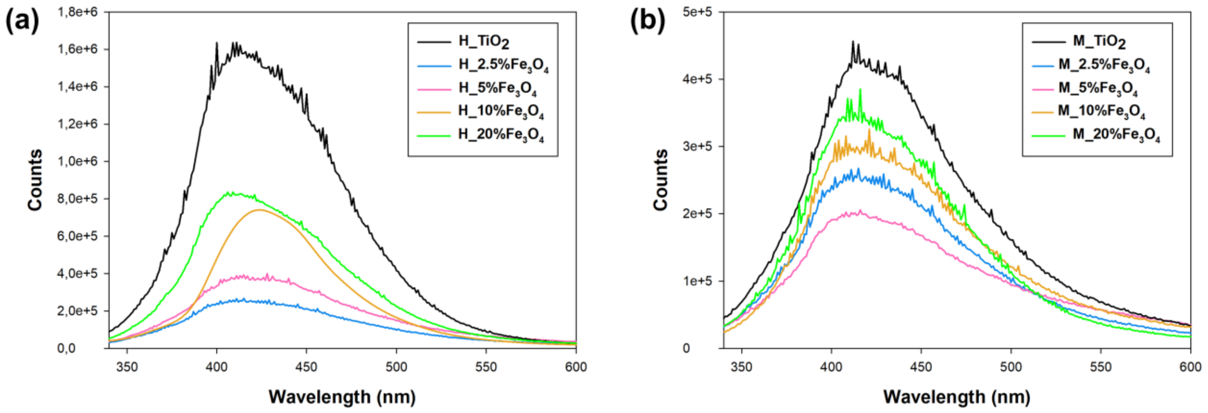

Fig. S3. The emission spectra for TiO_2-Fe_3O_4 systems synthesized by (a) conventional hydrothermal and (b) microwave methods.

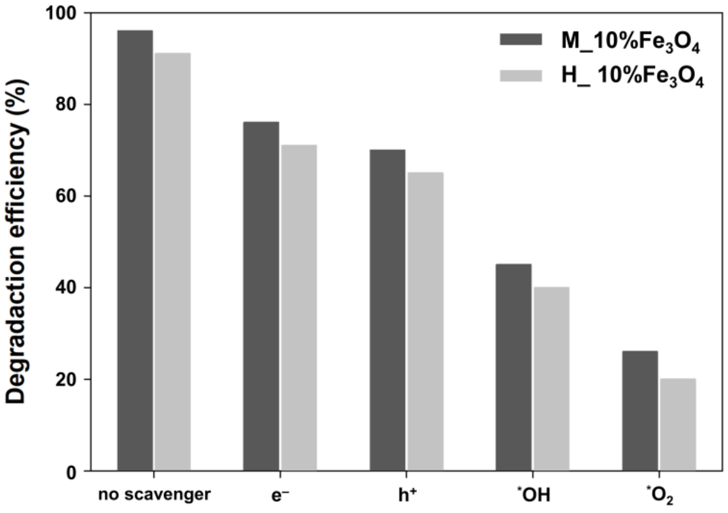

Fig. S4. The degradation efficiency of MNZ in the presence of radical scavengers.

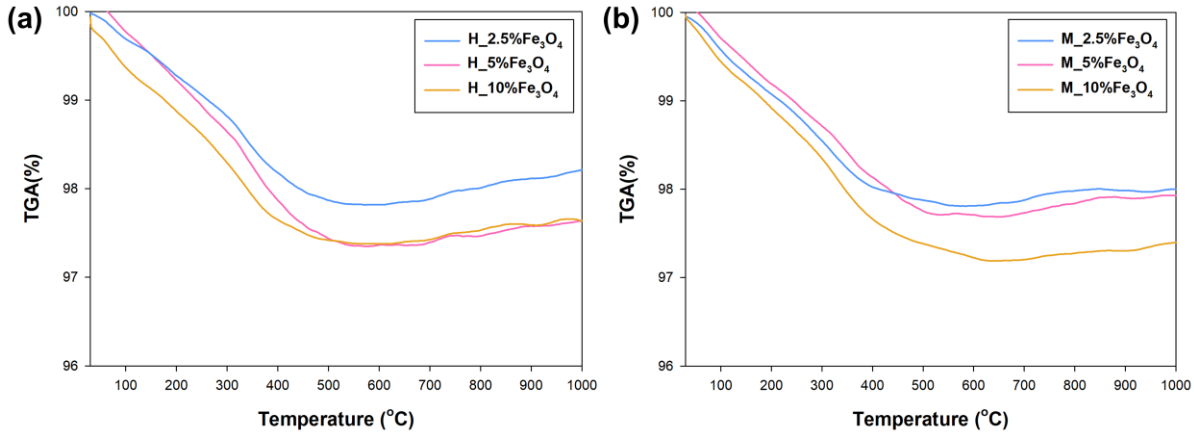

Fig. S5. The results of TGA analysis for TiO_2-Fe_3O_4 systems synthesized by (a) conventional hydrothermal and (b) microwave methods.

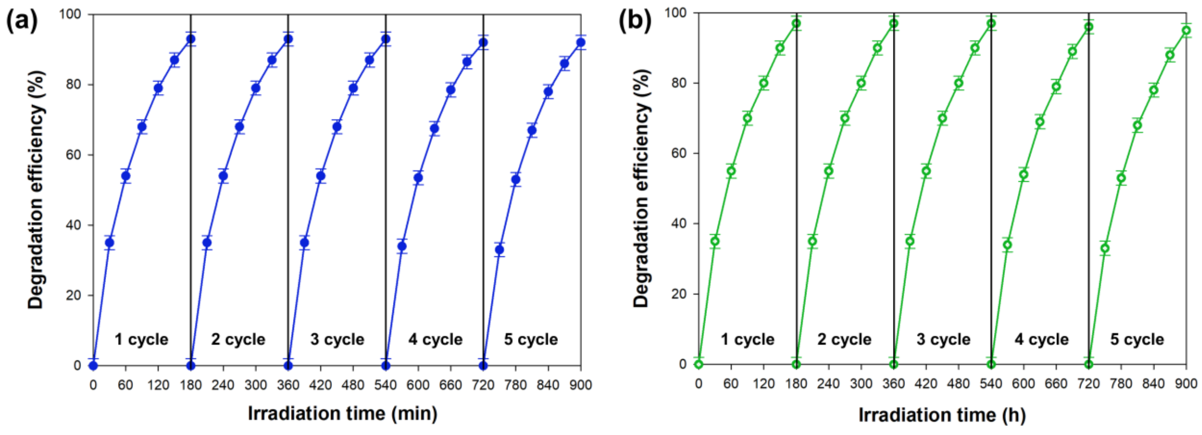

Fig. S6. Efficiency of MNZ photo-oxidation in the presence of (a) H_2.5%Fe_3O_4 and (b) M_2.5%Fe_3O_4 photocatalysts measured over five successive cycles.
